# Defect Detection of Subway Tunnels Using Advanced U-Net Network

**DOI:** 10.3390/s22062330

**Published:** 2022-03-17

**Authors:** An Wang, Ren Togo, Takahiro Ogawa, Miki Haseyama

**Affiliations:** 1 Graduate School of Information Science and Technology, Hokkaido University, N-14, W-9, Kita-ku, Sapporo 060-0814, Japan; 2 Faculty of Information Science and Technology, Hokkaido University, N-14, W-9, Kita-ku, Sapporo 060-0814, Japan; togo@lmd.ist.hokudai.ac.jp (R.T.); ogawa@lmd.ist.hokudai.ac.jp (T.O.); miki@ist.hokudai.ac.jp (M.H.)

**Keywords:** deep learning, semantic segmentation, U-Net, defect detection, subway tunnel

## Abstract

In this paper, we present a novel defect detection model based on an improved U-Net architecture. As a semantic segmentation task, the defect detection task has the problems of background–foreground imbalance, multi-scale targets, and feature similarity between the background and defects in the real-world data. Conventionally, general convolutional neural network (CNN)-based networks mainly focus on natural image tasks, which are insensitive to the problems in our task. The proposed method has a network design for multi-scale segmentation based on the U-Net architecture including an atrous spatial pyramid pooling (ASPP) module and an inception module, and can detect various types of defects compared to conventional simple CNN-based methods. Through the experiments using a real-world subway tunnel image dataset, the proposed method showed a better performance than that of general semantic segmentation including state-of-the-art methods. Additionally, we showed that our method can achieve excellent detection balance among multi-scale defects.

## 1. Introduction

With the growth of economies worldwide, various infrastructures such as tunnels, bridges, and viaducts have been constructed [1], which are indispensable for our daily life and are used by a large number of people in their daily lives [2]. However, infrastructures built more than five decades ago are experiencing aging problems, and the number of dilapidated infrastructures will significantly increase in the near future [1], and their maintenance cost will also increase exponentially [3,4]. Under these circumstances, a more efficient maintenance and management of infrastructures have become an urgent issue. Recently, much interest has been shown in smart maintenance and management technologies, including artificial intelligence (AI), Internet of Things (IoT), and big data analysis. These techniques have already been applied to real-world problems in various fields [5,6,7,8,9]. These techniques are required in the field of infrastructure to improve the efficiency and accuracy of infrastructure maintenance [10,11].

As an important infrastructure, urban railway systems have been mainly constructed during the high-speed economic growth period. In urban areas, the overground transportation network is already dense and its expansion potential is limited. On the other hand, the subway transportation environment, such as subway tunnels, is expected to expand further in the future. However, through the high frequency of use, tunnels that were built decades ago inevitably decay and suffer from a number of defects. Without repairs, these defects lead to significant economic losses and threaten safety.

In order to maintain a high level of security and economic growth, the daily maintenance and inspection of tunnels is necessary. Traditional inspection methods mainly rely on tunnel wall images taken by inspection vehicles or inspectors [12]. Inspectors look for deterioration such as cracks and leaks when taking images and the deterioration of tunnel walls is evaluated and repaired according to their conditions. This process is performed manually, and it takes much time and labor. Technologies that enable the automatic detection of defects are required to facilitate this process [13,14].

The standard strategy for supporting the inspection of subway tunnels is to construct a detector for the estimation of defects from tunnel wall images. Among all kinds of defects, automated crack detection has been studied for a long time, and various methods based on image processing have been proposed [15,16,17,18,19]. Recently, in the field of computer vision, the performance of image recognition has been significantly improved with the emergence of deep learning, which has been useful for various tasks [20,21,22,23,24]. Therefore, it is expected that image recognition technology will enable the development of a detector that can automatically identify defects in infrastructures.

Deep learning-based methods have achieved higher performances in detecting defect in infrastructures than traditional methods that use handcrafted image features [25,26,27]. However, when applying deep learning methods to real-world problems, various characteristics and situations have to be considered. Since there are various kinds of defects in subway tunnels such as cracks, cold joints, and leakages, existing deep learning methods cannot be directly applied to this task. Specifically, the following problems need to be addressed to improve detection performance:

**Problem 1:**
  Subway tunnel images have a high resolution and limited areas of defects. Hence, the problem of imbalance between the background and foreground in semantic segmentation is prominent.
**Problem 2:**
  Defects in subway tunnels have multi-scale variations. It is necessary to distinguish between these types since the repair action differs depending on the type of defect.
**Problem 3:**
  Subway tunnel image contains a complex background. Although there are no defects in the background area, it often contains structures similar to the defects due to the construction conditions.

Hence, it is desirable to devise more effective network architectures that can recover the details of defects in subway tunnel images and improve the detection accuracy of multi-scale defects.

To solve the above problems, we focus on the U-Net architecture [28], one of the most widely used methods in biomedical image segmentation tasks. The U-Net’s skip connection method, which can concatenate up-sampled feature maps with feature maps skipped from an encoder, makes it possible to effectively capture details and location information about objects. U-Net and its variants have achieved impressive segmentation results in computer vision tasks, especially in detecting multi-scale targets [29,30,31,32]. Because the cracks feature in our task is long and thin, we require the network to have the capacity to maintain the feature in high resolution; U-Net is a suitable choice for this. Specifically, the feature of cracks (small targets) is mainly captured by the high-resolution layer, and the water leakage feature is mostly captured by the low-resolution layer. Because of the succinct architecture, it is easy to add extra modules or change the architecture to improve the detection capacity for different kinds of segmentation targets in our task. The U-Net architecture is, therefore, suitable for our task.

In this paper, we propose an improved version of the U-Net architecture to solve the above problems. As a network design for the multi-scale target segmentation of a particular image dataset, the U-Net architecture is a suitable foundation network for our task. To solve Problem 1, we adjust the image dataset to balance background and foreground images to overcome the problem of background examples dominating gradients. To solve Problems 2 and 3, we optimize the network architecture using the following strategies: First, we replace all convolution blocks of the U-Net architecture with inception blocks [33]. Since the inception module consists of four different branches with different kernel sizes and enlarges the network’s receptive field, we can improve the network adaption to different scales of features. For our task, this improvement increases the capacity to detect multi-scale defects. In addition, for the same purpose, we replace the first convolution layer of the bridge layer with an atrous spatial pyramid pooling (ASPP) module from Deeplab-v2 [34]. Combining these two kinds of structures results in more precise detection and mitigates the over-fitting problem.

Our contributions are summarized as follows:

- We propose a novel advanced U-Net for defect detection using subway tunnel images.
- We design an architecture that can grasp the characteristics of a variety of defects. The experimental results show the effectiveness of our new architecture.

This paper is organized as follows: Summaries of related works on defect detection and classification are presented in Section 2. Next, Section 3 shows the data characteristics, and Section 4 shows the proposed method and the adopted network architectures. The experimental results are shown in Section 5. Finally, our conclusion is presented in Section 6.

## 2. Related Works

In this section, we discuss related works of computer vision tasks for application, U-Net family, and defect detection, respectively. Recent application tasks in computer vision are mentioned in Section 2.1, more specific architectures based on U-Net are explained in Section 2.2, and methods for defect detection are presented in Section 2.3.

### 2.1. Computer Vision Task for Application

Computer vision tasks have made great progress with the rise of deep learning technologies [35]. In the past, computer vision tasks have been studied mainly with the aim of recognizing objects in images; however, with the rise of deep learning, the level of accuracy close to real-world applications has been achieved [25,36]. The recognition level of general objects exceeded human accuracy in a competition held in 2015, and various methods for more advanced tasks such as object detection and pixel-level segmentation have been proposed [37,38]. In parallel, this technology has been applied not only in the field of computer science but also in various other fields. Transfer learning has shown that feature representations acquired by general image recognition can be useful for tasks in other domains [39,40]. In addition, a number of studies have been proposed for tasks where the amount of data is not sufficient [41].

Following general images, medical images are the next area where the technology is expected to be applied to society [42,43,44]. Medical images are highly specialized due to the clarification of imaging standards, but the quality of the captured images is high. Therefore, supervised learning, which is the speciality of deep learning, has succeeded in building relatively accurate models [45].

### 2.2. Deep Learning with U-Net and Its Variants

As a well-known biomedical image segmentation network, U-Net architecture in 2015 has a completely symmetric encoder–decoder structure, where features extracted from the same size convolutional layers are concatenated with corresponding up-sampling layers; thus, high- or low-level feature maps can be preserved and inherited by the decoder to obtain more precise segmentation. After that, its variants were proposed in the following years and are still applied to real-world segmentation tasks nowadays.

The common improved variants of U-Net are committed to redesigning convolutional modules and modifying down- and up-sampling. Namely, many varying methods such as TernausNet [46], Res-UNet [47], Dense U-Net [48], and R2U-Net [31] have been proposed. For example, TenausNet replaces the encoder part with VGG11, Res-UNet and Dense U-Net replace all submodules with res-connection and dense-connection, and R2U-Net combines recurrent convolution and res-connection as a submodule. U-Net++ [29] and U-Net 3+ [30] hope to increase multi-scale target detection capacity. The main advantage of these variants is that they can capture features of different levels and integrate them through feature superposition.

### 2.3. Defect Detection in Infrastructures

Before the high development age of deep learning techniques, the defect detection methods were mainly developed by using image processing method. In [10,11], the authors conducted surveys of newly developed robotic tunnel inspection systems and showed that they overcome these disadvantages and achieve high-quality inspection results. Additionally, Huang et al. reported a method for analyzing the morphological characteristics and distribution characteristics of structural damage based on an intelligent analysis method from visible images of tunnel images [13]. Furthermore, Koch et al. reported computer vision-based distress detection and condition assessment approaches related to concrete and asphalt civil infrastructure [49]. In addition, several methods for automatic detection based on computer vision techniques have been proposed [21,22]. Khoa et al. proposed automatic crack detection and classification methods by using morphological image processing techniques and feature extraction based on distance histogram-based shape descriptors [21]. Furthermore, Zhang et al. proposed a method called online CP-ALS to incrementally update tensor component matrices, followed by self-tuning a one-class support vector machine [24] for online damage identification [22].

In recent years, deep learning techniques have been successfully applied to defect detection tasks based on real-world datasets. For instance, Kim et al. [50] used Mask R-CNN to detect and segment defects in multiple kings of civil infrastructure. Bai et al. [51] used Robust Mask R-CNN for the task of crack detection. Specifically, they proposed a two-step method, called cascaded network, in which ResNet is used to classify defects and then some state-of-art segmentation networks are used. Huang et al. [52] proposed an integrated method, which combines a deep learning algorithm and Mobile Laser Scanning (MLS) technology, achieving an automated three-dimensional inspection of water leakages in shield tunnel linings. Choi et al. [53] proposed a semantic damage detecting network (SDDNet) for crack segmentation, which achieves real-time segmentation effectively negating a wide range of various complex backgrounds and crack-like features. Chen et al. [54] present a switch module to improve the efficiency of the encoder–decoder model, demonstrating it with U-Net and DeepCrack as examples. In this way, deep learning-based defect detection methods have shown promising results for the classification and segmentation tasks with the benefit of high representation ability.

## 3. Dataset

**Figure 1:**
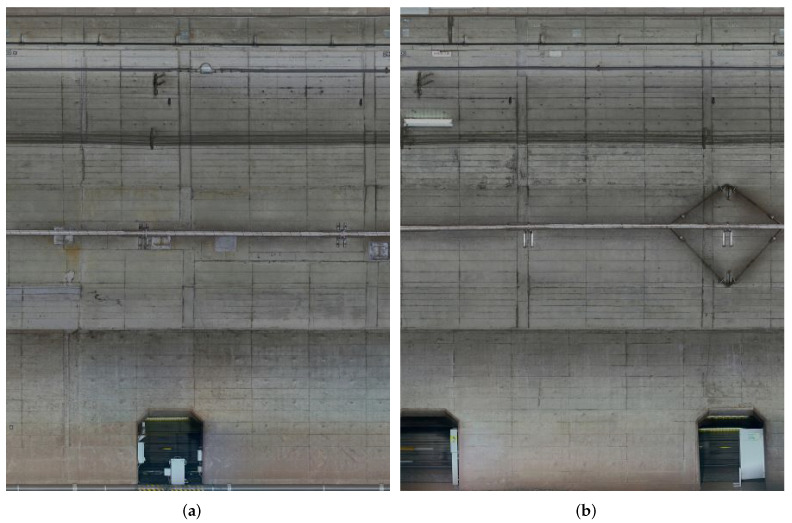
Examples of subway tunnel images used in this study. (**a**,**b**) are sample images taken from a visible camera for inspection. (Resolution: 1 mm/pixel, Image size: 12,088 × 10,000 pixels).

In this section, we explain the inspection data used in our study. Figure 1 shows examples of the subway tunnel image data. We can see that the tunnel image data have different characteristics of natural image data. The size of the images is approximately 12,088 × 10,000 pixels or 12,588 × 10,000 pixels and the resolution is 1 mm/pixel, and so they can be considered high-resolution images. Typically, analyzing high-resolution images requires enormous computer resources and such image sizes are not used in the input of deep learning models. On the other hand, the resizing process results in the loss of fine-scale defects. We solve this problem by the patch division processing.

**Figure 2:**
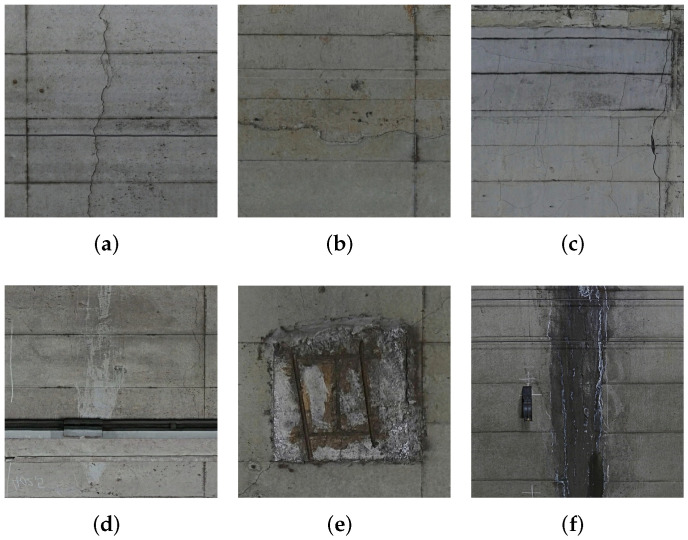
Example of defect images. (**a**–**f**) represent cracks, cold joint, construction repair, deposition, peeling, and trace of water leakage, respectively. (Resolution: 1 mm/pixel, Image size: 256 × 256 pixels).

The subway tunnel image data consist of defect and background images. Figure 2 shows defect patch examples divided from original images shown in Figure 2 (a) cracks, (b) cold joint, (c) construction repair (d) deposition (e) peeling, and (f) trace of water leakage. As shown in Figure 2, we can see that each type of defect has its characteristics such as different texture edges and color features. As for a two-class segmentation task, this intra-class variance will cause false alarms. For instance, the size and color of cracks (Figure 2a) are different from those of traces of water leakage (Figure 2f).

**Figure 3:**
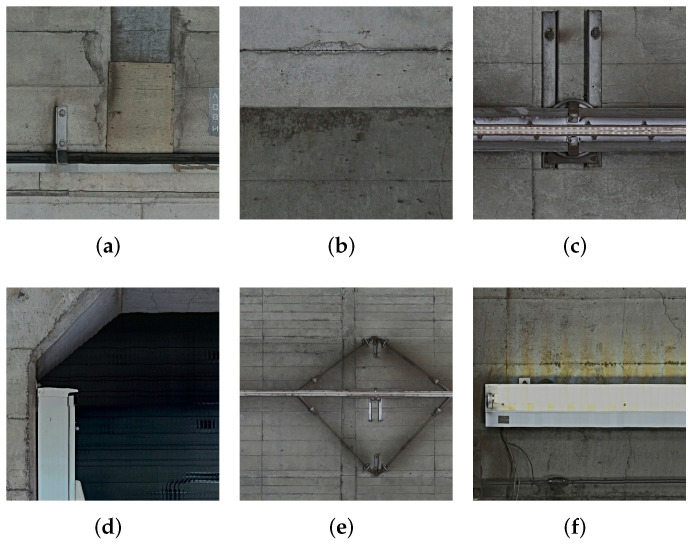
Example of background images. (**a**–**f**) show cable, concrete joint, connection component of overhead conductor rail, passage tunnels, overhead conductor rail, and lighter, respectively. (Resolution: 1 mm/pixel, Image size: 256 × 256 pixels).

Next, we show divided patch examples of background images that have no defects in Figure 3 (a) cable, (b) concrete joint, (c) connection component of overhead conductor rail, (d) passage tunnels (e) overhead conductor rail, and (f) lighter. In Figure 3, some of them have characteristics similar to those of defect images, which can also cause a serious false alarm problem.

## 4. Methodology

Inspired by Inception-v4, ASPP module, and U-Net, we propose a new model for defect detection. The proposed network combines the advantages of all three existing models. We explain data augmentation in Section 4.1 and introduce the architecture of our network in Section 4.2.

### 4.1. Data Augmentation

In this subsection, we propose our data augmentation strategy and patch selection method. First, we divide high-resolution subway tunnel images into multiple patches as shown in Figure 2 and Figure 3. Let $P_{i}\left(i=1,2,3,\ldots ,I\right)$ denote divided patches derived from the original images shown in Figure 1, where *I* represents the number of patches. Because of the imbalance distribution and multi-scale defects, we used an overlap strategy to ensure exhaustive defect patches, which extend the patch dataset. In addition, to construct the dataset via patch selection, we experimentally obtained a large-scale dataset containing background $B_{n}\;\left(n=1,2,\ldots ,N\right)$ and defect patches $D_{m}\;\left(m=1,2,\ldots ,M\right)$. Note that the ratio between *M* and *N* is approximately 7:3 and *N* + *M* = *I*.

For the training phase, since the dataset includes superfluous patches and a approximately half of them are background patches, it can cause a data imbalance problem. Under this condition, we randomly excluded some background patches to balance the number of patch samples. It should be noted that this strategy does not influence the detection accuracy. Finally, the ratio between defect and background patches can reach 1:1.

The advantage of data augmentation is that features between distributions of data can be resolved by pseudo-data generation. The model acquires a high degree of generality by learning to identify the transformed images as input. In recent years, this idea has been incorporated into self-supervised learning. In self-supervised learning, a transformation similar to data augmentation is performed, and learning is performed without labels. It has been reported that this method can dramatically improve the representational capability of the model itself. In this paper, we focus on data augmentation because we are interested in supervised learning.

### 4.2. Network Architecture

**Figure 4:**
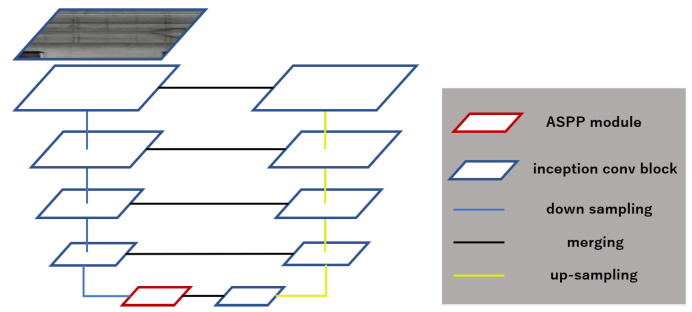
Overview of our defect detection network architecture.

**Figure 5:**
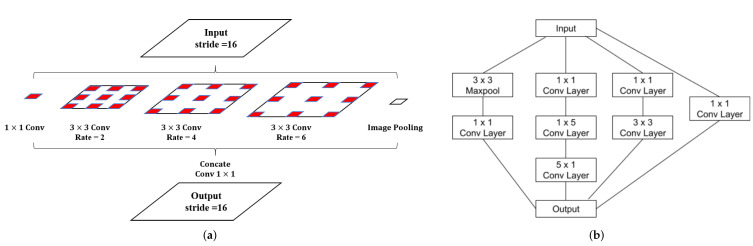
Modules introduced in our method. (**a**) represents the architecture of ASPP module and (**b**) represents the inception module.

In this subsection, we explain the network architecture used in our method. Figure 4 depicts a model architecture of the proposed method, and Table 1 represents the details of our network. We chose U-Net as our backbone model to achieve a high performance in the special data segmentation task. To increase the rate of detection of multi-scale defects in subway tunnel data, first, we replaced the convolution blocks of the U-Net architecture with inception blocks modified from Inception-v3 as shown in Table 1. Inception blocks extend the feature capture area to increase accuracy and mitigate the over-fitting problem. Second, we added the ASPP module to our model, and we imitated the usage of the ASPP in Deeplab-v3+ to set it after the last layer of the encoder (the bridge layer, middle of the network) shown in Figure 5a. In shallow architectures, the size of the encoder’s last layer is no less than 16 × 16. We adjusted the parameter settings of multiple parallel atrous convolutions in the ASPP module for adaptation to our task. In the following, we explain the details of our model.

Our network consists of stacked layers of modified inception blocks shown in Figure 5b in the U-Net-based encoder–decoder network. The inception blocks consist of four parallel branches. Three of them have convolution layers with different kernel sizes, and the last one has one max-pooling layer. We replaced the 5 × 5 convolution layer with 5 × 1 and 1 × 5 convolution layers to decrease the training parameters. In the original U-Net architecture, the encoder part contains 8 convolution blocks. In addition, the output of every 2 convolution blocks is down-sampled by a max-pooling layer, and to construct a deeper network, we add one inception block before each max-pooling layer, increasing the total number of convolution operations in the encoder from 8 to 12.

At the end of the encoder part, we replaced the bridge’s first convolution layer with the ASPP module, which is shown in Figure 5a; the input was split into 5 equal partitions. In the original ASPP module, the atrous rates of three 3 × 3 convolutions were set to 6, 12, and 18 (with 256 filters and batch normalization) to adapt to the input size, which is over 37 × 37. When the rate value is close to the feature map size, the 3 × 3 filter degenerates to a 1 × 1 filter, and the atrous convolution loses its effectiveness. In our task, the input size was limited to 256 × 256 pixels, and after 4 max-pooling operations, the final input size of the ASPP module became 16 × 16, which is less than the required 37 × 37. Therefore, we changed the atrous rates from 4, 8, and 16 to 2, 4, and 6 to adapt to the input size. After the ASPP module, a 1 × 1 convolution operation (with 1024 channels) was added to merge the bridge layer.

In the decoder part, we used a convolution transpose layer (with a kernel size of 3 × 3 and a stride size of 2) to perform the up-sampling operation. Instead of using the deeper architecture as the encoder, we replaced all basic convolution layers with inception blocks.

## 5. Experiments and Results

This section shows quantitative and qualitative evaluations to confirm our network’s effectiveness for detecting defects in subway tunnel images. The experimental settings are explained in Section 5.1, and the results and discussion are presented in Section 5.2 and Section 5.3, respectively. Experimental data were provided by Tokyo Metro Co., Ltd, a Japanese subway company.

### 5.1. Settings

In our experiments, 47 images made up the subway tunnel image dataset. The images were obtained from visible cameras with high resolutions (e.g., 12,088 × 10,000 pixels or 12,588 × 10,000 pixels), and we divided the images into multiple patches of 256 × 256 pixels with a sliding interval of 64 pixels.

In the training phase, we filtered the patches using the strategy introduced in Section 4.1. The pixel-ground truth of defects was determined by inspectors. We selected 280,000 patches from 29 images as our training dataset. In this dataset, the ratio between the background and defect patches was set to 1:1. Then, in the validation phase, seven images were divided by the same strategy, as in the training phase, and finally, 71,818 patches were selected. The last 11 images were used in the test phase. We only used the same dividing strategy without abandoning background patches. Therefore, the number of patches used in the test phase was 326,172, which is significantly larger than that in the training phase. After the test phase, we generated estimation images by recombining the estimation results and the average probability of each pixel.

For the semantic segmentation task, Recall, Precision, F-measure, and Intersection over Union (IoU) were used to evaluate the binary classification performance as our estimation metrics. They can be calculated as follows:

(1) $$\begin{array}{cc} Recall & =\frac{\mathrm{TP}}{\mathrm{TP}+\mathrm{FN}}, \end{array}$$

(2) $$\begin{array}{cc} Precision & =\frac{\mathrm{TP}}{\mathrm{TP}+\mathrm{FP}}, \end{array}$$

(3) $$\begin{array}{cc} \text{F-measure} & =\frac{2\times \mathrm{Recall}\times \mathrm{Precision}}{\mathrm{Recall}+\mathrm{Precision}}, \end{array}$$

(4) $$\begin{array}{cc} IoU & =\frac{\mathrm{TP}}{\mathrm{TP}+\mathrm{FP}+\mathrm{FN}}, \end{array}$$

where TP, TN, FP, and FN represent the number of true-positive, true-negative, false-positive, and false-negative samples, respectively.

We compared our method with classic segmentation methods including Deeplab-v3+ (CM1) [55], FCN (CM2) [56], and SegNet (CM3) [57]. Since the input of the network was set to 256 × 256, the output size of the encoder in Deeplab-v3+ was 16 × 16. According to our method, we adjusted the parameter settings of multiple parallel atrous convolutions in the ASPP module using the same strategy as introduced in Section 4.2. In addition, since our network is based on the U-Net architecture, we added several previous U-Net versions as comparative methods (CM4-CM7). The design of each method is shown in Table 2. Among them, CM5 [58] added additional down-sampling blocks to both the encoder and decoder of the network, changing the down-sampling stride from 16 to 32.

### 5.2. Results

In this subsection, we show the evaluation results and discuss some important details of the proposed model.

#### 5.2.1. Quantitative Analysis

Table 3 shows the detection rate of all defects. From Table 3, we can compare the defect detection performance of our method and comparative methods (CM1-CM7). In these metrics, IoU, which is the standard metric of the semantic segmentation field, is the most important value to evaluate the total performance. We can see that PM obviously outperformed all CMs in this metric.

Next, Table 4 shows the recall rate of detection of each defect. From Table 4, we can observe the specific defect detection performance of our method and comparative methods (CM1-CM7). It should be noted that the metric Recall was used for the evaluation of each defect detection performance since the small crack defects were directly included. For the evaluation of the detection performance of cracks, IoU is not the best evaluation metric because of the difficulty of pixel-level matching. Moreover, considering the application situation, over-detection is considered preferable to miss-detection for the detection of defects. From the above reasons, we selected the evaluation metric Recall in this evaluation.

The proposed method outperforms all comparative methods. According to Table 3 and Table 4, we can further discuss the importance of each component.

**Limitation of Deeplab-v3+ (CM1)**:
  Deeplab-v3+ used atrous convolution, ASPP module, and a simplified decoder branch, achieving great improvement compared with the baseline. There was a slight difference in the detection accuracy for various kinds of defect. Although Deeplab-v3+ applied multiple kinds of modules to improve detection performance for multi-scale defects, it still lacks detection accuracy for large-scale defects as shown in Table 3.
**FCN and SegNet (CM2, CM3)**:
  FCN and SegNet, as classic segmentation networks, show a certain degree of incompatibility in our subway tunnel dataset, not only with a low accuracy but also with a large number of false detection instances as shown in Table 3. Especially, the performance of SegNet is extremely poor. Although the detection accuracy of small targets such as cracks can be maintained, it is almost impossible to detect large defects as shown in Table 4. These result in the low overall detection accuracy and precision of the network. Unlike U-Net, the SegNet decoder uses the max-pooling indices received from the corresponding encoder to perform nonlinear upsampling of the input feature map as a typical symmetric encoder–decoder architecture. It is considered that this function did not work well in the subway tunnel dataset.
**Effectiveness of ASPP module (CM4)**:
  In CM4, this module increases F-measure from 0.428 to 0.444 and IoU from 0.272 to 0.286 compared with the baseline module (CM7) in Table 3. Additionally, the obtained results from Table 4 suggest that the addition of the ASPP module significantly improved the detection performance of small-, medium-, and large-scale defects. The obtained results show the effectiveness of the ASPP module.
**Effectiveness of layer extend operation (CM5)**:
  In CM5, compared with the baseline (CM7), this module increases F-measure from 0.428 to 0.495 and IoU from 0.272 to 0.329 as shown in Table 3. Additionally, Table 4 suggests that CM5 is superior to CM4, CM6, and the baseline (CM7). These results suggest that deeper networks improve the detection of all scales of defects. However, this operation could not be applied to networks with the ASPP module due to patch size limitations in the experimental setting.
**Effectiveness of Inception module (CM6)**:
  In CM6, we only replaced all convolution blocks with the inception module. This operation increased F-measure from 0.428 to 0.443 and IoU from 0.272 to 0.285 compared with the baseline (CM7) in Table 3. Additionally, Table 4 shows that the detection rate of each scale significantly improved compared with the baseline. This indicates that the addition of the inception module can contribute to the representation ability of low- and high-level information.
**Analysis of the proposed method**:
  As shown in Table 3, PM outperformed all other methods. Furthermore, from Table 4, we can see that PM achieves better accuracy in detecting large-scale defects but has some limitations in detecting small-scale defects. The limitation of small-scale defects may influence the detection performance of the inspection task. Thus, qualitative analysis is also required.

#### 5.2.2. Qualitative Analysis

**Figure 6:**
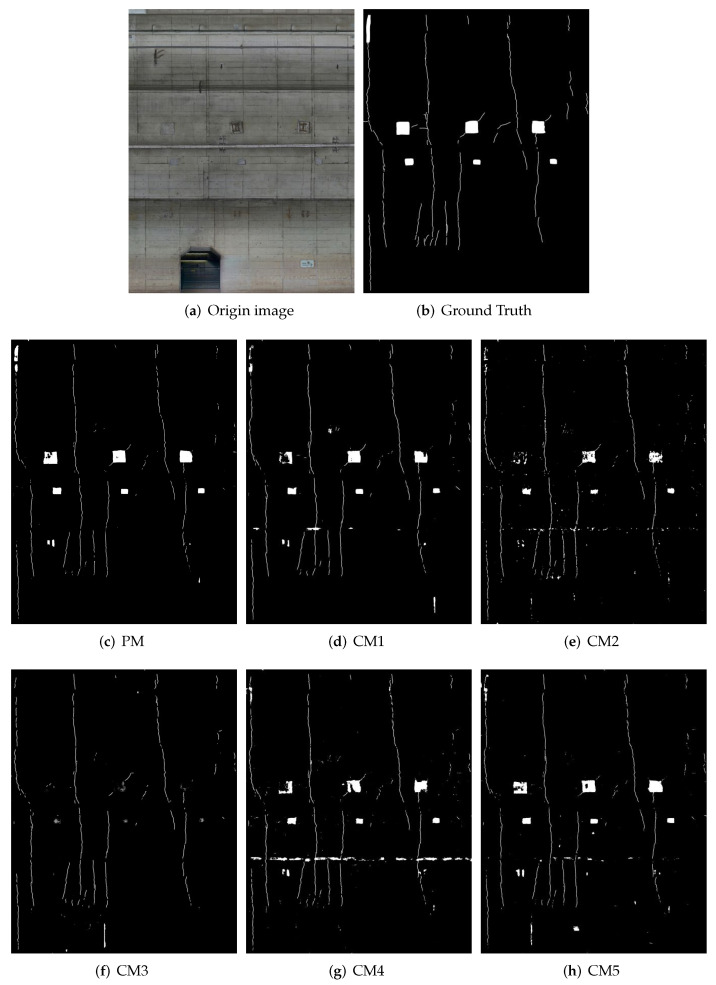

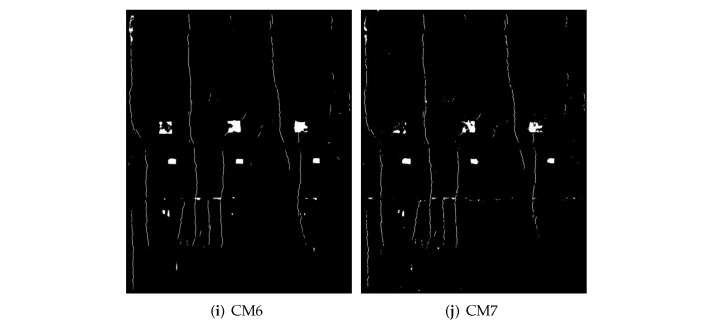
Results of proposed method and comparative methods. (From left to right: (**a**): original image; (**b**): ground truth; (**c**): results obtained by the proposed method; and (**d**–**j**): results obtained by the comparative methods.)

**Figure 7:**
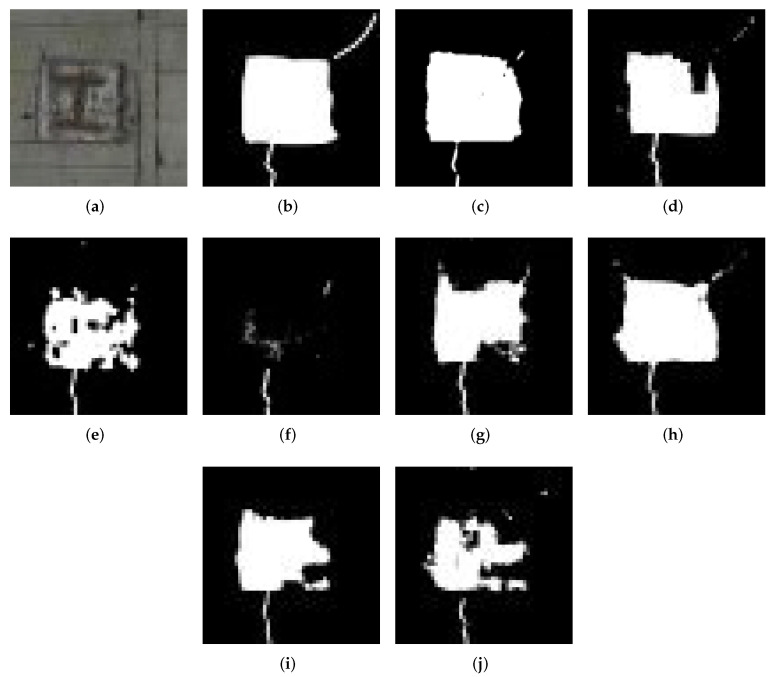
Example of the result in peeling detection. (**a**) Original image. (**b**) Ground Truth. (**c**) PM. (**d**) CM1. (**e**) CM2. (**f**) CM3. (**g**) CM4. (**h**) CM5. (**i**) CM6. (**j**) CM7.

**Figure 8:**
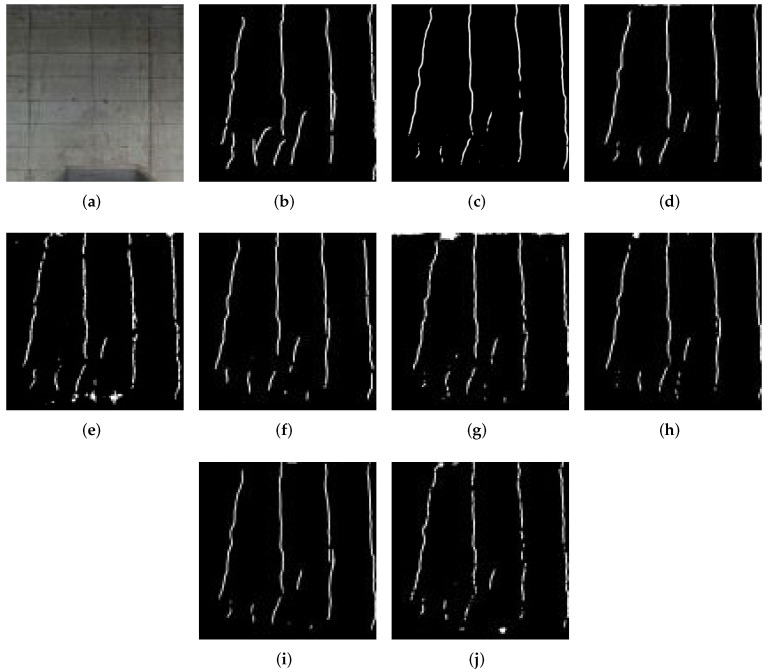
Example of the result for crack detection. (**a**) Original image. (**b**) Ground truth. (**c**) PM. (**d**) CM1. (**e**) CM2. (**f**) CM3. (**g**) CM4. (**h**) CM5. (**i**) CM6. (**j**) CM7.

**Figure 9:**
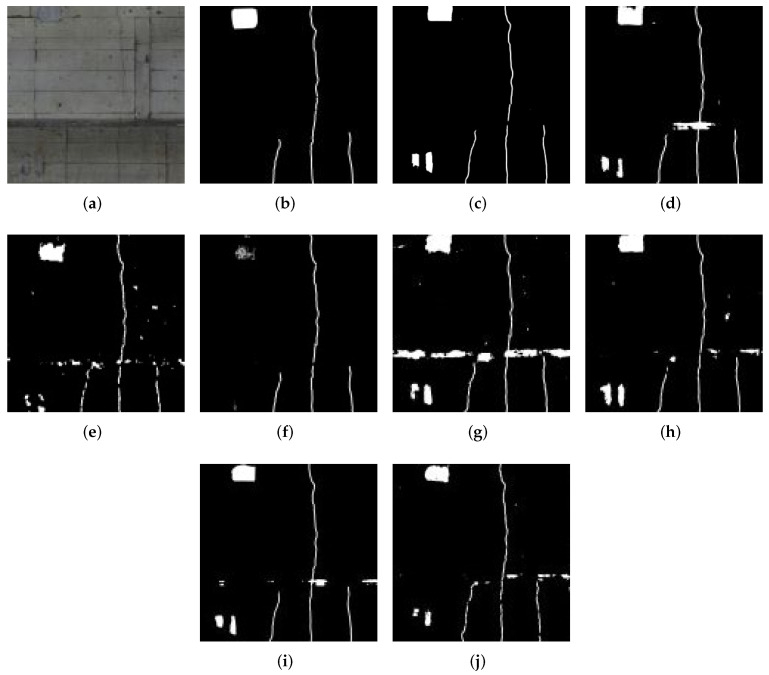
Example of the results of over-fitting parts. (**a**) Origin image. (**b**) Ground truth. (**c**) PM. (**d**) CM1. (**e**) CM2. (**f**) CM3. (**g**) CM4. (**h**) CM5. (**i**) CM6. (**j**) CM7.

In this part, we discuss the visual quality of the results. The estimation results are shown in Figure 6, Figure 7, Figure 8 and Figure 9. Figure 6 shows detection result samples of all regions of the test image. Figure 7 and Figure 8 show the detection results of peeling and cracks. From Figure 6, Figure 7 and Figure 8, we can see that PM achieves a high detection quality when detecting various defects compared to CMs. On the other hand, we show the over-fitting result sample in Figure 9. In some cases, we observed that vertical cracks tend to over-fit in our model. The quantitative analyses show that the proposed method has some limitations in detecting small-scale defects, and according to Figure 8, these limitations may not influence the actual inspection works. Compared with all CMs, the result of PM achieves fewer instances of false detection, which would lead to less unnecessary work for inspectors.

### 5.3. Discussion

In the field of image recognition, various models have been proposed consistently owing to the AI boom. In the models for general object recognition, the error rate of recognition now exceeds that of humans, and there is a glimpse of a direction to target more advanced tasks. Applications of AI are beginning to be explored in all areas, one of which is infrastructure maintenance. In this paper, we have proposed a method for detecting defects in subway tunnel images. By constructing a model that takes into account the characteristics of the data, the proposed method achieved a higher accuracy in detecting defects compared to conventional methods.

What we should consider here is how much the system should achieve to reach the accuracy that can be applied in the real world. The quantitative evaluation results obtained from this experiment showed that the IoU was around 0.3–0.4. This value may not be sufficient when compared to the accuracy of general image recognition. However, as shown in the results of the qualitative evaluation, cracks and other defects in the image can be detected even if there is some deviation. For example, if we consider the practical applications of the proposed method, such as supporting the registration of defects in CAD systems or identifying dense regions of defects, we can say that the proposed method has reached a system that can be applied in practice.

There are some limitations in this study. This study was conducted using data from a certain subway line in Japan, and there is still room for future studies on the general applicability to a wide variety of data. In this study, 47 high-resolution subway tunnel images were divided into patches to enable the network training; however, it would be desirable to have a larger number of images to verify the robustness of our method. In addition, since the accuracy is considered to vary depending on the year of construction of tunnels, verification using a wide variety of data is necessary. Specifically, the condition of the wall depends on the construction method of the subway tunnel, and furthermore, the new construction method may be completely different from the conventional construction method. When considering the versatility of the model, it will be necessary to verify the versatility of the model for various types of data.

## 6. Conclusions

In this study, we present a new version of the U-Net architecture to improve the detection performance of defects in subway tunnel images. By introducing ASPP and inception modules in the U-Net-based network architecture, we improved the capacity of the network for defect detection. The experimental results on a real-world subway tunnel image dataset showed that our method outperformed other segmentation methods quantitatively and qualitatively. Different from conventional crack detection methods, our model can detect various types of defects in a single model, which enhances the practicality for supporting tunnel inspections. In future works, we will investigate a new strategy for enhancing detection accuracy and discuss its application to other real-world tasks.

## Figures and Tables

**Table 1:** Architecture of the proposed model.

Type	Size/Stride	Output Size	Depth
Inception Module	3 × 3/1	256 × 256 × 64	3
Inception Module	3 × 3/1	256 × 256 × 64	3
Inception Module	3 × 3/1	256 × 256 × 64	3
Max Pooling	3 × 3/2	128 × 128 × 64	1
Inception Module	3 × 3/1	128 × 128 × 128	3
Inception Module	3 × 3/1	128 × 128 × 128	3
Inception Module	3 × 3/1	128 × 128 × 128	3
Max Pooling	3 × 3/2	64 × 64 × 128	1
Inception Module	3 × 3/1	64 × 64 × 256	3
Inception Module	3 × 3/1	64 × 64 × 256	3
Inception Module	3 × 3/1	64 × 64 × 256	3
Max Pooling	3 × 3/2	32 × 32 × 256	1
Inception Module	3 × 3/1	32 × 32 × 512	3
Inception Module	3 × 3/1	32 × 32 × 512	3
Inception Module	3 × 3/1	32 × 32 × 512	3
Max Pooling	3 × 3/2	16 × 16 × 512	1
The ASPP module	–	16 × 16 × 1024	2
Inception Module	3 × 3/1	16 × 16 × 1024	3
Deconvolution	3 × 3/2	32 × 32 × 512	3
Cat	–	32 × 32 × 512	1
Inception Module	3 × 3/1	32 × 32 × 512	3
Inception Module	3 × 3/1	32 × 32 × 512	3
Deconvolution	3 × 3/2	64 × 64 × 256	1
Cat	–	64 × 64 × 512	1
Inception Module	3 × 3/1	64 × 64 × 256	3
Inception Module	3 × 3/1	64 × 64 × 256	3
Deconvolution	3 × 3/2	128 × 128 × 128	1
Cat	–	128 × 128 × 256	1
Inception Module	3 × 3/1	128 × 128 × 128	3
Inception Module	3 × 3/1	128 × 128 × 128	3
Deconvolution	3 × 3/2	256 × 256 × 64	1
Cat	–	256 × 256 × 128	1
Inception Module	3 × 3/1	256 × 256 × 64	3
Inception Module	3 × 3/1	256 × 256 × 64	3
Sigmoid	1 × 1/1	256 × 256 × 1	1

**Table 2:** Differences in the proposed method (PM) and U-Net-based comparative methods (CM4-CM7) used in the experiment.

Method	Inception	ASPP	Layer Extend
PM	✓	✓	-
CM4	-	✓	-
CM5	-	-	✓
CM6	✓	-	-
CM7 (Baseline)	-	-	-

**Table 3:** Defect detection performance of the proposed method (PM) and the comparative methods (CMs).

Method	Recall	Precision	F-Measure	IoU
PM	0.660	0.436	0.525	0.356
CM1 [55]	0.564	0.375	0.451	0.291
CM2 [56]	0.494	0.315	0.385	0.238
CM3 [57]	0.410	0.136	0.204	0.158
CM4	0.493	0.405	0.444	0.286
CM5	0.532	0.463	0.495	0.329
CM6	0.617	0.346	0.443	0.285
CM7	0.588	0.336	0.428	0.272

**Table 4:** Recall of all kinds of defects in each method.

Defect	Recall							
	PM	CM1	CM2	CM3	CM4	CM5	CM6	CM7
Peeling	0.921	0.866	0.729	0.191	0.795	0.905	0.711	0.655
Floating	0.802	0.711	0.568	0.199	0.708	0.782	0.651	0.533
Crack (0.3 mm–0.5 mm)	0.173	0.230	0.163	0.209	0.159	0.140	0.125	0.110
Crack (0.5 mm–1 mm)	0.358	0.385	0.430	0.334	0.407	0.382	0.361	0.326
Crack (1 mm–2 mm)	0.402	0.463	0.384	0.422	0.455	0.434	0.409	0.388
Crack(2mm+)	0.414	0.409	0.394	0.431	0.467	0.444	0.426	0.389
Cold joint	0.013	0.017	0.016	0.014	0.016	0.016	0.007	0.005
Honeycomb	0.084	0.251	0.230	0.010	0.030	0.210	0.090	0.080
Patching (intermediate pile)	0.819	0.734	0.616	0.159	0.721	0.816	0.656	0.591
Alligator crack	0.362	0.308	0.216	0.063	0.317	0.368	0.306	0.244
Early construction repair	0.423	0.375	0.271	0.061	0.394	0.504	0.306	0.297
Deposition	0.054	0.049	0.015	0.001	0.080	0.012	0.005	0.010
Construction repair	0.591	0.307	0.167	0.078	0.413	0.556	0.364	0.375

## Data Availability

Not applicable.
